# A Postsynaptic Role for Short-Term Neuronal Facilitation in Dendritic Spines

**DOI:** 10.3389/fncel.2016.00224

**Published:** 2016-09-30

**Authors:** Sunggu Yang, Mariton D. Santos, Cha-Min Tang, Jae Geun Kim, Sungchil Yang

**Affiliations:** ^1^Department of Nano-Bioengineering, Incheon National UniversityIncheon, South Korea; ^2^Department of Neurology and Department of Physiology, University of Maryland School of MedicineBaltimore, MD, USA; ^3^Office of Dietary Supplement Programs, Center for Food Safety and Applied Nutrition, US Food and Drug AdministrationCollege Park, MD, USA; ^4^Division of Life Sciences, College of Life Sciences and Bioengineering, Incheon National UniversityIncheon, South Korea; ^5^Department of Biomedical Sciences, City University of Hong KongKowloon, Hong Kong

**Keywords:** synaptic integration, sensory perception, dendritic spike, NMDA receptor, short-term synaptic plasticity

## Abstract

Synaptic plasticity is a fundamental component of information processing in the brain. Presynaptic facilitation in response to repetitive stimuli, often referred to as paired-pulse facilitation (PPF), is a dominant form of short-term synaptic plasticity. Recently, an additional cellular mechanism for short-term facilitation, short-term postsynaptic plasticity (STPP), has been proposed. While a dendritic mechanism was described in hippocampus, its expression has not yet been demonstrated at the levels of the spine. Furthermore, it is unknown whether the mechanism can be expressed in other brain regions, such as sensory cortex. Here, we demonstrated that a postsynaptic response can be facilitated by prior spine excitation in both hippocampal and cortical neurons, using 3D digital holography and two-photon calcium imaging. The coordinated action of pre- and post-synaptic plasticity may provide a more thorough account of information processing in the brain.

## Introduction

Prior information is retrieved and read out while being integrated with incoming signals (Larson and Lynch, 1986; Körding and Wolpert, 2004; Köver and Bao, 2010; Hasselmo and Stern, 2014; Howard et al., 2014). The dynamic modification of the incoming signals in the brain relies heavily on past experience. In fact, a response facilitation in a temporal sequence is common in the *in vivo* condition (Calford and Semple, 1995; Brosch and Schreiner, 1997, 2000; Rosen and Mooney, 2003; Wehr and Zador, 2005; Kuo and Wu, 2012). Paired-pulse facilitation (PPF) observed with *in vitro* brain slices has been suggested as a potential synaptic mechanism of *in vivo* response facilitation in a sequence (Brosch and Schreiner, 2000; Regehr, 2012). In PPF, a prior electrical pulse enhances a neuronal response to a following pulse as a result of enhanced probability of glutamate release in the presynaptic terminal; thus PPF largely involves a presynaptic role in this response facilitation.

Even before presynaptic PPF was conceptualized, the effect of a prior input on postsynaptic responses had been investigated (Larson and Lynch, 1986). This study suggested that NMDA receptors (NMDARs) could cause a prolongation of the postsynaptic response when membrane depolarization precedes synaptic input. Recently, the NMDAR-dependent mechanism for response facilitation has been revisited in hippocampus, namely short-term postsynaptic plasticity (STPP or dendritic hold and read; Santos et al., 2012; Yang et al., 2014). Upon synaptic input, the “glutamate-bound but Mg^2+^ blocked” state of postsynaptic NMDARs persists for a period in individual dendrites. The prior information stored in this electrically silent or weak state (or “priming”) can then be conditionally read out as a regenerative dendritic spike triggered (or “gated”) by subsequent neuronal excitability. Such a mechanism holds two characteristics critical for effective signal processing: (1) it is a biophysical mechanism whereby information of prior glutamate exposure can be stored for short periods of time; and (2) it is a cellular mechanism for robust signal amplification.

Despite the potential significance of such STPP for information processing, it’s precise mechanism and subcellular localization has not been adequately demonstrated at the level of the dendritic spine of cortical neurons. Here, we used various activation paradigms and imaging tools in brain slices to demonstrate a postsynaptic role of short-term synaptic plasticity in dendritic spines. These findings help establish a more complete understanding of synaptic integration and plasticity in sensory perception.

## Materials and Methods

### Brain Slice Preparation

All animal handling procedures were approved by the Institutional Animal Care and Use Committee of the University of Maryland, Incheon National University, and City University of Hong Kong. Animals were treated in accordance with the *National Institutes of Health* Guide for the care and use of laboratory animals, and the *Animal Welfare Act (7* U.S.C. et seq.). Sprague-Dawley rats and C57BL/6 mice (postnatal age: 3–6 weeks) for brain slices were deeply anesthetized with halothane. The brains were quickly removed and placed into chilled (4°C), oxygenated (5% CO_2_ and 95% O_2_) slicing medium containing (in mM): 212 sucrose, 5 KCl, 1.23 NaH_2_PO_4_, 26 NaHCO_3_, 11 glucose, 1.5 MgCl_2_, 2.5 CaCl_2_. Transverse slices (300 μm) were cut normal along the septo-temporal plane. Brain slices was then transferred to a holding chamber containing oxygenated physiological saline made up of (in mM): 124 NaCl, 4 KCl, 1.23 NaH_2_PO_4_, 26 NaHCO_3_, 10 glucose, 1.5 MgCl_2_, 2 CaCl_2_. Also, brain slices of primary auditory cortex were prepared according to our established method (Yang et al., 2011b). After ~1 h recovery, individual slices were transferred to a recording chamber. Oxygenated physiological saline was continuously superfused at a rate of 1.5 ml/min at 32–33°C temperature.

### Whole-Cell Patch Recording

Whole-cell patch recordings were obtained using an Axon instruments Axoclamp 700B Amplifier (Molecular Devices), and recording pipettes had tip resistances of 3–7 MΩ when filled with a solution containing (in mM): 135 K-gluconate, 5 KCl, 1 MgCl_2_, 0.02 CaCl_2_, 0.2 EGTA, 10 HEPES, 4 Na_2_-ATP, 0.3 Na-GTP. Alexa 594 (50 μM) was included in the internal solution for visualization. The pH and osmolarity of intracellular solution were adjusted to 7.3 and 290 mOsm, respectively. pClamp Version 10.2 software (Molecular Devices) or Igor Pro (WaveMetrics) was used for data acquisition. Hyperpolarizing current pulses (10–20 pA; 0.5 s duration; 5–10 s interval) were continuously applied to monitor cells’ input resistance (Rin). During recordings, the access resistance was often monitored; it was typically 20–30 MΩ. Recordings were excluded if Rin changed by >15%.

### 3D Digital Holography Uncaging

The procedures for digital holographic photolysis have been described (Yang et al., 2011a). The holographic beam was brought into the optical axis of an upright fluorescence microscope (Olympus BX51) below the epi-fluorescence unit, with a long-pass dichroic mirror. The output beam of a 150 mW, 405 nm diode laser (CNI Laser) was expanded by a beam expander (3×) to fill the short axis of a reflective spatial light modulator (SLM; LCOS Hamamatsu, model X10468–05). The SLM plane was projected onto the back aperture of the microscope objective through a telescope (L1, f1 = 750 mm; L2, f2 = 500 mm). The magnification of the telescope was chosen in order to match the SLM short axis with the diameter of the objective’s back aperture (Olympus, 60×, W 0.9 NA). The undiffracted component (zero order spot) was removed by placing a small (<0.5 mm) anodized metal plate on antireflective coated glass plate at the focal plane of L1. The algorithm for the phase hologram calculation and calibration of the temporal spatial resolution were previously described (Yang et al., 2011a). MNI-caged-L-glutamate was prepared fresh each day at final concentration in physiological solution. Glutamate uncaging pulse duration was set to 0.5 ms. Tetrodotoxin (TTX; 1 μM) was added to examine synaptic responses over a wider range of input intensity except when investigating a role for intrinsic excitability as a gating signal.

**Figure 1:**
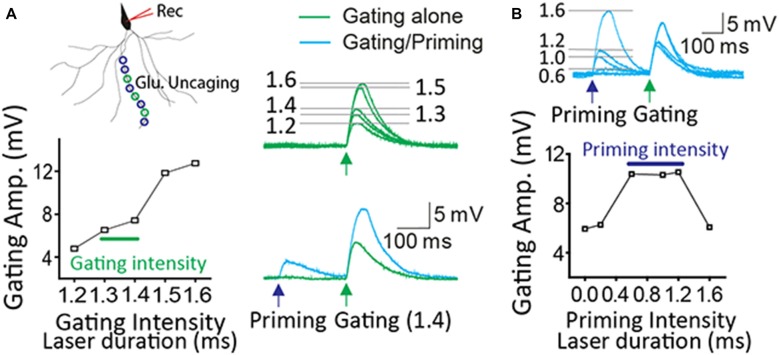
**Optimization of priming and gating intensity. (A)** The priming stimulation produced the potentiated read-out response when gating stimulation was set up just prior to a dendritic spike. **(B)** There are a certain range of priming signals to generate a read-out response efficiently. In this case, priming intensity (0.6~1.2 ms laser duration) produced the facilitation when gating intensity ranged from 1.3 to 1.4 ms.

The input-output curve of gating and priming responses was tested before STPP experiments as previously established (Santos et al., 2012). The proper intensity of a gating signal was determined based on the input-output curve of gating responses. The laser power (or duration) just before laser intensity giving rise to a dendritic spike was chosen for gating intensity (Figure 1A, 1.3~1.4 ms in this case). Once a dendritic spike occurred, its amplitude usually became saturated. The laser power of priming intensity was adjusted into a level to potentially induce a dendritic spike upon the given gating input. A wide range of priming intensity (0.6~1.2 ms in this case) produced the read-out response efficiently (Figure 1B). This scheme was also applied to experiments with two-photon imaging.

### Two-Photon Imaging

A two-photon imaging system (Prairie Technologies) was used as described previously (Yang et al., 2014, 2016). Briefly, A Chameleon Ultra II laser (Coherent) was tuned to 810 nm for Ca^2+^ imaging. Epi- and trans-fluorescence signals were captured through a 60×, 1.4 NA oil immersion condenser (Olympus). Fluorescence was split into red and green channels using dichroic mirrors and band-pass filters (T560LPXR, ET525/50, ET620/60; Chroma). Green fluorescence (G, Fluo-5F) was captured on an H10770PA-40 photomultiplier tube (PMTs, Hamamatsu). Red fluorescence (R, Alexa 594) was captured with an R9110 PMT. Data were presented as averages of 10 events per site, and expressed as Δ(G/R)/(G/R)_sat_*100 (simply, ΔG/G_sat_), where (G/R)_sat_ was the maximal fluorescence in saturating (2 mM) Ca^2+^. The other laser split from a Ca^2+^ imaging laser path was tuned to 720 nm for uncaging of MNI-caged-L-glutamate. Glutamate uncaging pulse duration was set to 0.5 ms. All experiments were done at ~32.5°C temperature.

### Chemicals

*Fluo-5F* and *Alexa Fluor 594* were from Molecular Probes. *MNI-caged-L-glutamate, Ifenprodil, Ro 25-6981* and *TTX* were purchased from Tocris (Ellisville, MO). *NVP-AAM077* was from Sigma. Imaging dyes and *MNI-caged-L-glutamate*/blockers were introduced to the pipette and the artificial cerebrospinal fluid, respectively.

### Statistics

All data are shown as mean ± standard error (SEM). An analysis of variance (ANOVA) of Fisher’s PLSD *post hoc* test was performed for between-group comparison, while a paired *t*-test was done for within-groups (significance, *P < 0.05; **P < 0.01).

## Results

### STPP on a Hippocampal Basal Dendrite

**Figure 2:**
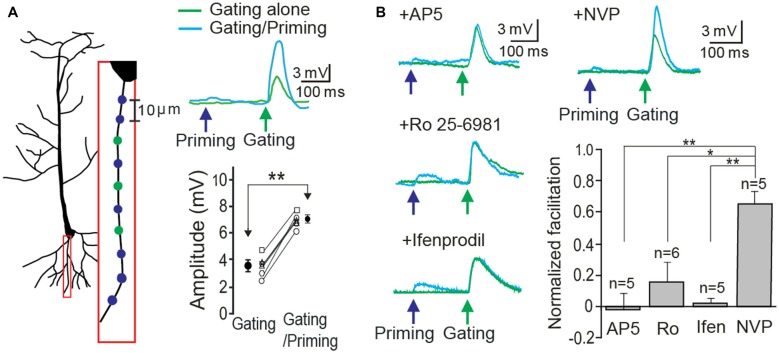
**Short-term postsynaptic plasticity (STPP) on a hippocampal dendrite. (A)** Illustration of a CA1 pyramidal neuron and priming (blue)/gating (green) glutamate uncaging spots in a basal dendrite (left). The representative responses to priming plus gating (cyan trace) and gating alone (green trace) glutamate uncaging were shown as photoactivated excitatory postsynaptic potentials (EPSPs). Read-out facilitation was observed when a priming photolysis proceeded a gating photolysis (*n* = 6 cells). **(B)** Neuronal facilitation was completely blocked by *AP5* (an NMDAR blocker), *Ro 25-6981* and *Ifenprodil* (NR2B blockers), while it remained by *NVP-AAM077* (an NR2A blocker). Error bars represent SEM. *p < 0.05; **p < 0.01.

In order to demonstrate STPP on a single basal dendrite, whole cell patch recordings were made on CA1 neurons in hippocampal slices. *Alexa 594* (20 μM) in the patch electrode was dialyzed into the dendritic arbor. When a fluorescence signal of a basal dendrite became visible, photolysis sites on spine areas of the dendrite were identified and targeted according to our established method (Yang et al., 2014, 2015). Two sets of photo-stimulation using 3D digital holography were employed to a single dendrite separated temporally and spatially. *TTX* and *MNI-caged-L-glutamate* were employed to identify a postsynaptic component of synaptic responses. A priming signal was created by glutamate uncaging to multiple synaptic locations over ~100 μm length (blue circles and cyan trace on the blue arrow, Figure 2A). Subsequently, a gating stimulus was directed at neighboring spots on the same dendrite 200 ms later (green circles and trace on the green arrow, Figure 2A). The gating response was significantly facilitated when preceded by a priming stimulus in all six cells tested (Figure 2A, gating: 3.61 ± 0.30 mV vs. gating + priming: 6.83 ± 0.25 mV, paired *t*-test, *p* < 0.001, *n* = 6 cells). Such facilitation was completely blocked by the application of an NMDAR antagonist, *AP5* (1 μM), an NR2B (an NMDAR subunit) antagonist, *Ifenprodil* (1 μM) and *Ro 256981* (1 μM), but not an NR2A antagonist, *NVP-AAM077* (0.5 μM) which was known to preferentially block the NR2A subunit of NMDARs at this concentration (Frizelle et al., 2006; Bartlett et al., 2007). With the same gating intensity, facilitation under various antagonists was normalized with that of each control case (Figure 2B, AP5: −0.02 ± 0.10; *Ro 25-6981*: 0.16 ± 0.13; *Ifenprodil*: 0.02 ± 0.03; *NVP-AAM077*: 0.66 ± 0.10, Fisher’s PLSD *Post hoc* test, *p < 0.05; **p < 0.01). This result confirmed our previous demonstration that postsynaptic facilitation can largely occur through an NR2B-dependent mechanism.

**Figure 3:**
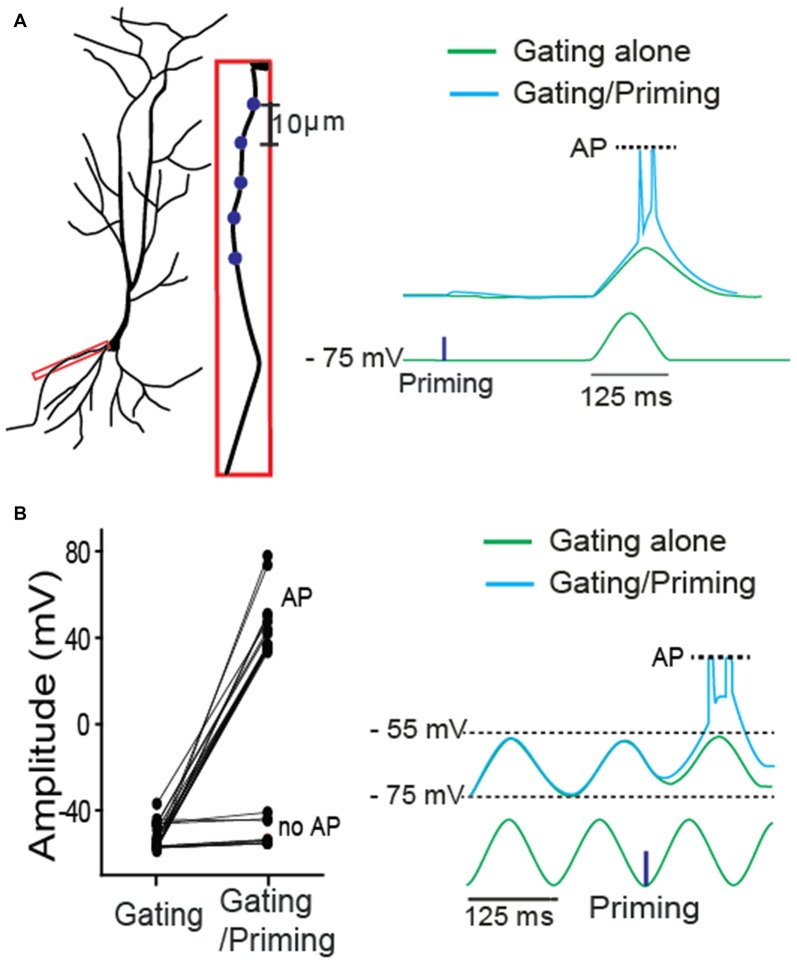
**A role of intrinsic membrane excitability as a gating signal in STPP. (A)** Blue dots for priming photostimulation were positioned in a basal dendrite. The read-out action potentials (APs) were observed only when intrinsic excitability was combined with the prepositioned priming photolysis. Blue dots for priming photostimulation were located in a basal dendrite. **(B)** ~8 Hz oscillatory sinusoidal wave elicited by current injection triggered APs only when the peak was preceded by a priming signal (uncaging). The all-or-none APs were shown with 71% success rate (AP: *n* = 17; no AP: *n* = 7).

Next, we asked whether, in addition to synaptic input, membrane excitability can serve as a gating signal. A single sinusoidal wave for mimicking membrane fluctuation was elicited by injecting a biased sine wave current, while a priming signal was photostimulated on a basal dendrite (Figure 3A). Action potentials (APs) were triggered only when the membrane fluctuation was coupled with the preceded priming input. Then, a gating signal was generated by injecting a sinusoidal current into the soma to mimic an oscillatory sinusoidal wave (~8 Hz, a frequent rhythmic form in CA1 hippocampus) while a priming signal was photostimulated on dendritic spines. APs were triggered only when the membrane fluctuation was coupled with the priming signal in the absence of TTX (cyan trace, Figure 3B). They were triggered with 71% success rate when the photolysis-induced priming signal co-occurred a postsynaptic depolarization (Figure 3B, 5 neurons of 3 animals, AP, 17; no AP, 7). These data suggest that the gating signal can be read out by either synaptic or intrinsic excitability irrespective of the location of input sources.

### Hippocampal STPP at the Spine Level

**Figure 4:**
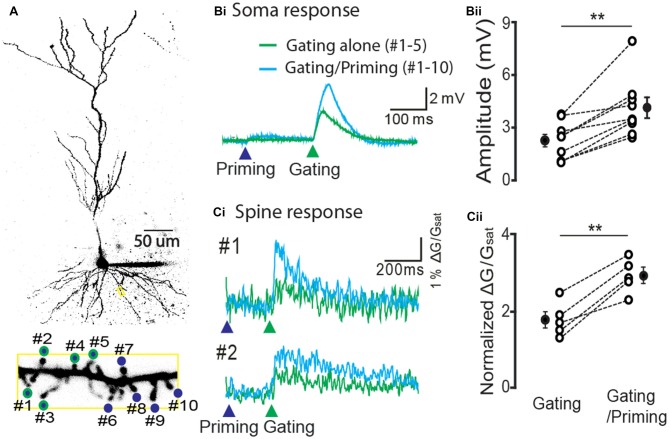
**Hippocampal STPP at a spine level. (A)** Two photon image of a CA1 pyramidal neuron and its basal dendrite with priming (blue color)/gating glutamate uncaging spots (green outline) on the individual spines in a basal dendrite. **(B,C)** The representative responses to priming plus gating (cyan trace) and gating alone (green trace) glutamate uncaging were shown as photoactivated EPSPs. When the priming stimulus proceeded the gating stimulus, the facilitated read-out responses (cyan traces) were elicited in both the electrical recording at the cell body **(Bi)** and calcium transients on individual spines **(Ci)**. Population data in electrical recording on cell body (**Bii**, *n* = 9 cells/4 animals) and calcium transients on the spines (**Cii**, *n* = 5 cells/3 animals). Error bars represent SEM. **p < 0.01.

In order to test the expression of STPP at the spine level, we stimulated individual spines of a CA1 pyramidal neuron using the two-photon calcium imaging method. We identified spines of a basal dendrite that were co-labeled with *Alexa 594* (20 μM) and a dynamic Ca^2+^ indicator *Fluo 5F* (100 μM, Figure 4A). For a priming stimulus, 10 spines of a basal dendrite (blue circles) were targeted for photolysis, while a gating stimulus targeted five of these spines (green outlines). The gating stimulus alone produced an electrical response in the soma (the green trace on the green arrow head, Figure 4Bi, *n* = 9 cells) and Ca^2+^ transients on the spines (the green trace on the green arrow head, 5 cells among 9 cells, Figure 4Ci). When a priming stimulus preceded the gating stimulus, both the electrical and calcium signals were remarkably facilitated (Figure 4Bii, gating alone: 2.3 ± 0.35 mV vs. gating/priming: 7.17 ± 0.73 mV, paired-*t* test, *p* < 0.001, *n* = 9; Figure 4Cii, normalized ΔG/G_sat_ of gating alone: 1.78 ± 0.21 vs. gating/priming: 2.94 ± 0.20, paired-*t* test, *p* < 0.001, *n* = 5). This result suggests that STPP is a response integrated by dynamic activity of individual spines.

### Cortical STPP

**Figure 5:**
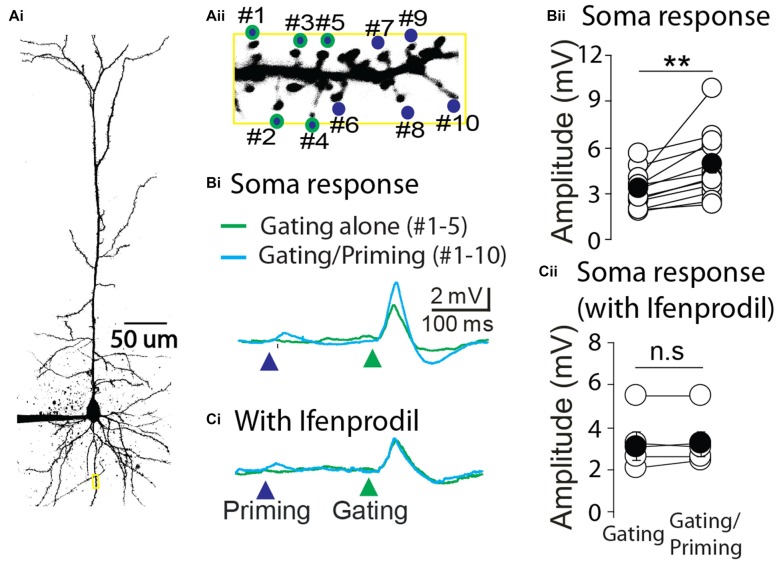
**Cortical STPP. (A)** Two photon image of a pyramidal neuron **(Ai)** and its basal dendrite with priming (blue color)/gating (green outline) glutamate uncaging spots on the individual spines in a basal dendrite **(Aii)**. **(B)** The representative responses to priming plus gating (cyan trace) and gating alone (green trace) glutamate uncaging were shown as photoactivated EPSPs. When the priming stimulus preceded the gating stimulus, the facilitated response (cyan trace) was elicited in the electrical recording at the cell body **(Bi)**. **(C)** NR2B selective blocker, Ifenprodil, completely blocked the neuronal facilitation **(Ci)**. Population data of control (**Bii**, *n* = 12 cells/4 animals) and Ifenprodil (**Cii**, *n* = 5 cells/4 animals) in electrical recording of the cell body. **p < 0.01; n.s, *P* > 0.05.

Next we asked whether STPP is present in other brain areas. To this end, layer 4 pyramidal neurons of primary auditory cortex were tested with the same experimental strategy as CA1 pyramidal neurons. Ten spines of a cortical neuron were identified and targeted to photolysis of MNI-glutamate (Figure 5A). Similar to the results from hippocampal neurons, the electrical responses in the soma were remarkably facilitated when a priming stimulus preceded the gating stimulus (Figures 5Bi,ii, gating alone: 3.3 ± 0.35 mV vs. gating/priming: 4.86 ± 0.62 mV, paired *t* test, *p* < 0.001, *n* = 12). The facilitation was completely blocked by the application of an NR2B selective blocker, *Ifenprodil* (1 μM; Figures 5Ci,ii, gating alone: 3.10 ± 0.63 mV vs. gating/priming: 3.20 ± 0.59 mV, paired *t* test, *p* > 0.05, *n* = 5). These results implicate that NMDAR-dependent activity within individual spines mediate STPP in cortical neurons.

## Discussion

In the past, studying STPP has been difficult due to limitation in recording techniques. With the advent of biochemical activation and imaging tools including 3D digital holography and two-photon microscopy, we demonstrate here the existence of STPP in hippocampus and sensory cortex. The information storage mechanism is present in dendritic spines. Such information held for a short period of time can facilitate gating signals such as fluctuations in synaptic and intrinsic excitability, suggesting the important role on sensory signal processing.

### The Role of STPP in Sensory Information Processing

Response facilitation in a temporal sequence has been previously suggested as a core model of feature detection and discrimination (Calford and Semple, 1995; Brosch and Schreiner, 1997, 2000; Fortune and Rose, 2000; Wehr and Zador, 2005; Schreiner and Polley, 2014). PPF tested with *in vitro* brain slices has been suggested as a potential synaptic mechanism of the observed *in vivo* response facilitation (Brosch and Schreiner, 2000). However, the causal relationship between *in vitro* PPF and *in vivo* response facilitation still remains speculative. It is noteworthy that the NMDAR-dependent STPP is similar to the *in vivo* response enhancement phenomenon in light of the temporal scale (~600 ms) and all-or-none expression pattern (Brosch and Schreiner, 2000; Yang et al., 2014). Hence, we propose that STPP acts along with PPF as a cellular process underlying sensory perception.

### Prerequisites for STPP

STPP is determined by a combination of several factors such as dendritic morphology, the spatiotemporal pattern of synaptic inputs and active intrinsic conductances. STPP was observed when a priming and gating stimulus were co-localized on the same distal dendrite. By contrast, it was not observed when one of two stimuli was separately directed on an apical trunk or adjacent dendrite and two inputs were separated by more than 50 μm even on the same dendrite (Santos et al., 2012). At the spine level, however, we showed that priming and gating synaptic inputs can be neighboring ones for STPP induction as in Figure 2A. Also, a gating stimulus of rhythmic membrane depolarization (which does not necessarily share the same synapses with the priming stimulus) consistently induced STPP, showing independence of input sources (Figure 3). It is notable that different input patterns of a gating stimulus, whether spatially distributed or clustered within a single dendrite, were capable of producing facilitation, which is consistent with a previous study (Losonczy and Magee, 2006). Taken together, these data provide evidence that facilitation likely occurs only when a priming and gating input are situated on the same dendrite unless they are not far apart from each other within the dendrite. Consistent with our findings, there is physiological evidence that single dendrites are tuned to different orientations of object movement in pyramidal neurons of visual cortex (Jia et al., 2010).

STPP can be induced by its characteristic regenerative Ca^2+^ influx through NMDARs (i.e., NMDA spikes). Although the basal level of a synaptic event is largely initiated by non-NMDAR-mediated cationic currents, its amplification begins with NMDA spikes in thin dendrites where most excitatory synaptic events occur. An earlier synaptic input (“priming stimulus”) may not produce a depolarization large enough to remove the Mg^2+^ block of the NMDARs. However, when a subsequent stimulus (“gating stimulus”) which by itself would produce only a modest depolarization is delivered to the same dendrite, its response can be markedly facilitated by promoting Mg^2+^ liberation and initiating an NMDA spike. The question then arises whether STPP can be a false-positive facilitation due to prolonged dendrite depolarization following a priming input (Milojkovic et al., 2004). However, the 200 ms interval between two stimuli, in our study, was sufficient to prevent voltage and calcium deflections from overlapping. Priming responses were fully recovered into baseline as shown in both somatic recording and spine calcium imaging (Figure 4). Also, STPP was induced even at longer intervals up to 600 ms which could guarantee temporal separation (Yang et al., 2014). These results support the STPP mechanism of the “glutamate-bound but Mg^2+^ blocked” state rather than electrical interference by prolonged dendritic depolarization. Those physiological characteristics of STPP are distinct from those of the PPF with respect to location of action (PPF, presynaptic glutamate release vs. STPP, postsynaptic NMDARs), activation pattern (PPF, graded vs. STPP, NMDA spike-driven all-or-none) and spatial integration spectrum (PPF: synapse-specific vs. STPP, dendrite-specific).

### The Neuronal Structure for STPP

Pyramidal neurons are connected to one another in an associational fashion along the longitudinal axis (e.g., CA1-CA1 connection) in hippocampus (Yang et al., 2014) and also along the representation of stimulus features (e.g., isofrequency corticocortical line) in cortex (Imig and Reale, 1980; Lee and Winer, 2008; Oswald and Reyes, 2008; Brown and Hestrin, 2009). The connectivity of the pyramidal-pyramidal neurons and their biophysical properties (holding and amplifying electrical traces) allow signals to pass through a linearly connected brain circuit. Considering that neurons receive streams of information in a temporal sequence, the brain must have a capacity to process a temporal sequence of information for a short period of time; the individual information cannot be properly interpreted without a form of short-term mnemonic buffer. Signals bearing temporal information (e.g., priming, including subthreshold responses) can be, for example, read out by pronounced neuronal activity (i.e., gating, including a certain frequency of brain oscillation), and subsequently converted into a spatial pattern of information along the linear circuit (Hasselmo and Stern, 2006, 2014; Santos et al., 2012). This signal conversion of time-to-space may be necessary for object recognition in the brain.

## Author Contributions

Sunggu Yang, MDS, C-MT and Sungchil Yang designed experiments. Sunggu Yang, MDS, JGK and Sungchil Yang performed the experiments and prepared the figures. Sunggu Yang and Sungchil Yang wrote the main manuscript text. All authors reviewed the manuscript.

## Conflict of Interest Statement

The authors declare that the research was conducted in the absence of any commercial or financial relationships that could be construed as a potential conflict of interest.
